# New Progress in Early Diagnosis of Atherosclerosis

**DOI:** 10.3390/ijms23168939

**Published:** 2022-08-11

**Authors:** Heyu Meng, Jianjun Ruan, Zhaohan Yan, Yanqiu Chen, Jinsha Liu, Xiangdong Li, Fanbo Meng

**Affiliations:** 1 Jilin Provincial Precision Medicine Key Laboratory for Cardiovascular Genetic Diagnosis, Jilin Provincial Cardiovascular Research Institute, Jilin University, Changchun 130033, China; 2 Jilin Provincial Engineering Laboratory for Endothelial Function and Genetic Diagnosis of Cardiovascular Disease, Jilin Provincial Cardiovascular Research Institute, Jilin University, Changchun 130033, China; 3 Jilin Provincial Molecular Biology Research Center for Precision Medicine of Major Cardiovascular Disease, Jilin Provincial Cardiovascular Research Institute, Jilin University, Changchun 130033, China

**Keywords:** coronary atherosclerosis, computed tomography coronary angiography, genes, protein, trace element

## Abstract

Coronary atherosclerosis is a potentially chronic circulatory condition that endangers human health. The biological cause underpinning cardiovascular disease is coronary atherosclerosis, and acute cardiovascular events can develop due to thrombosis, platelet aggregation, and unstable atherosclerotic plaque rupture. Coronary atherosclerosis is progressive, and three specific changes appear, with fat spots and stripes, atherosclerosis and thin-walled fiber atherosclerosis, and then complex changes in arteries. The progression and severity of cardiovascular disease are correlated with various levels of calcium accumulation in the coronary artery. The therapy and diagnosis of coronary atherosclerosis benefit from the initial assessment of the size and degree of calcification. This article will discuss the new progress in the early diagnosis of coronary atherosclerosis in terms of three aspects: imaging, gene and protein markers, and trace elements. This study intends to present the latest methods for diagnosing patients with early atherosclerosis through a literature review.

## 1. Introduction

Coronary atherosclerosis is a life-threatening chronic cardiovascular condition. Coronary atherosclerosis is one of the leading causes of death among the aged. The localized deposition of fat in the arteries, along with the development of smooth muscle cells and a fibrous matrix, is the primary issue with atherosclerosis. Over time, this encourages the formation of atherosclerotic plaques [1]. The biological root of cardiovascular disease is atherosclerosis, and thrombosis, platelet aggregation, and unstable atherosclerotic plaque rupture will result in arterial stenosis or occlusion, resulting in acute cardiovascular illness [2,3]. Because inflammation plays a major part in all stages of coronary atherosclerosis’s progression, it is commonly regarded as a chronic inflammatory disease. Inflammation is the common cause of the physiological and pathological alterations that occur throughout the onset and progression of coronary atherosclerosis. Years of extensive research have revealed that coronary atherosclerosis has a complicated etiology, with lipid buildup and chronic inflammation in the artery wall being the crucial attributes [4].

Typically, atherosclerosis of the coronary arteries is linked with alterations in lipid metabolism and hypercholesterolemia [5]. Increased low-density lipoprotein (LDL) levels are known cardiovascular disease risk factors [6]. However, the pathophysiology of the disease appears to be more complex than alterations in lipid metabolism, involving numerous variables, with inflammation being the most significant [7]. Local endothelial dysfunction, which may be induced by blood flow instability near an artery’s bend or bifurcation, is the pathological cause of the development of atherosclerosis. The activation of vascular endothelial cells in response to mechanical stress results in the recruitment of circulating immune cells. An atherosclerotic plaque is developed by circulating monocytes adhering to and infiltrating into the affected area of the artery wall, differentiating into macrophages, aggressively taking up lipids through phagocytosis, and producing a significant number of foam cells [8].

Fat spots and stripes, atherosclerosis, thin-walled fiber atherosclerosis, and eventually complicated arteries are the three types of particular changes that develop in sequence as atherosclerosis progresses [9,10]. According to the disease’s course, the American College of Cardiology divides them into six groups [11,12]. The Type I and Type II early phases can be identified by lipid patches. Yellow patches and a few foam cell accumulations can be seen in the artery’s intima. Lipid droplets and smooth muscle cells that T lymphocytes have penetrated are present in the intima. Preplaque, or Type III, is characterized by more extracellular lipid droplets generating lipid nuclei between the layers of smooth muscle cells in the intima and mesomembrane without forming a lipid pool. The stage of atherosclerotic plaque production is Type IV. Since the lipids are more concentrated, the lipid pool has already formed. The artery wall is distorted, and the intimal structure is obliterated. The development of thin-walled fibro-atheroma is a hallmark of Type V. It is the lesion of atherosclerosis that is most recognizable. Lumen stenosis develops when white plaque enters the artery lumen. A proliferative fibrous cap encircles the lipid pool, and the intima of the plaque surface is obliterated. Type VI is referred to as a complicated atherosclerotic lesion, which is a serious lesion. It is distinguished by bleeding, necrosis, ulceration, calcification, and fibrous plaque wall thrombosis.

**Figure 1:**
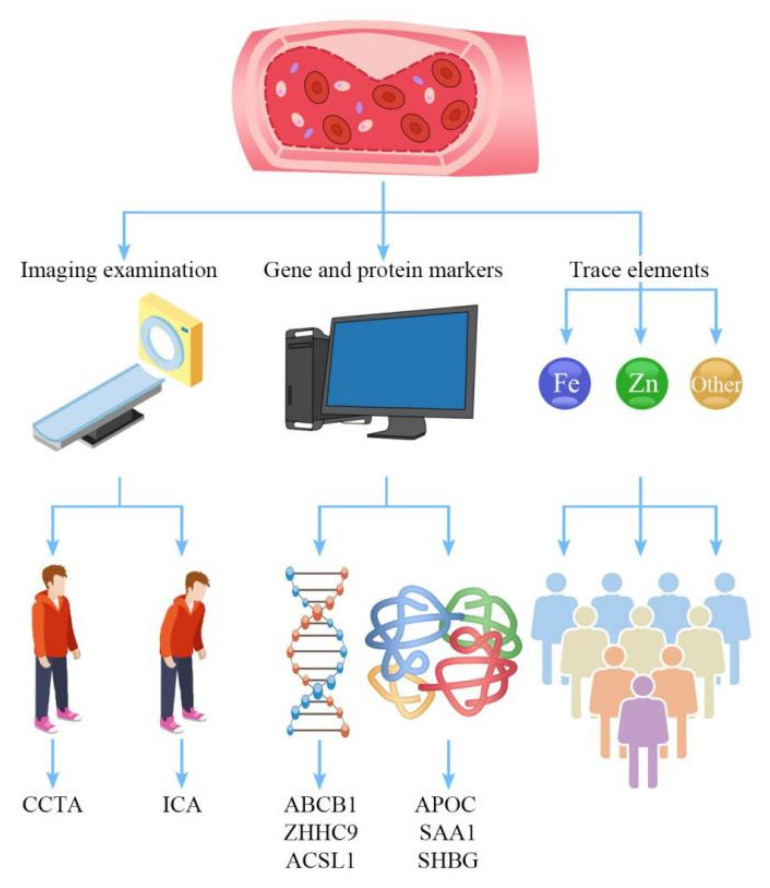
Flow chart of the three examination modes of coronary atherosclerosis. From left to right are imaging examination, gene and protein marker examination, and trace element examination.

Calcification is a key cause of coronary atherosclerosis [13] and a good marker to forecast future heart problems. Heart disease worsens and spreads at different rates depending on how much calcium builds up in the body [14]. Coronary atherosclerosis is treated and has a favorable prognosis when the amount and extent of calcification are determined early [15]. The purpose of the present study is to discuss the new progress in the early diagnosis of coronary atherosclerosis in terms of three aspects: imaging, gene and protein markers, and trace elements (see Figure 1 for details).

## 2. Imaging Examination

High-spatial-temporal-resolution invasive coronary angiography (ICA) is the gold standard for examining coronary lumens [16,17,18]. Over the last three decades, computed tomography coronary angiography (CCTA) has evolved into an effective and inexpensive imaging tool for assessing coronary artery disease (CAD). Because normal CTCA images have a strong negative predictive value, they can effectively eliminate substantial CAD, minimizing the requirements for additional imaging tests and lowering ICA use in patients with low and intermediate CAD risk [19,20,21,22]. Because of its cost-effectiveness and clinical efficacy, the National Institutes of Health guidelines in 2016 advised that CCTA be used as a first-line survey in all suspected stable CAD patients [23]. The Society of Cardiovascular Computed Tomography’s steering committee developed acceptable standards for using CCTA to guide doctors [24].

CCTA is widely used to identify (a) patients with indicative coronary heart disease who have a low or moderate pre-test probability of coronary heart disease and (b) patients with a low or moderate pre-test probability of coronary heart disease who have newly diagnosed heart failure and no known ischemic heart disease, as well as (c) in the evaluation of cardiac health before surgery in patients thought to have a low or moderate risk of coronary heart disease. The risk factors for atherosclerosis include smoking, older age, diabetes, high cholesterol, and hypertension. As mentioned earlier, these are the fundamental elements, and a person with these risk factors will undoubtedly have a higher probability of developing coronary heart disease. These risks serve as the foundation to determine which patients should undergo CCTA when determining whether their risks are high or low. Several studies have demonstrated that CCTA provides patients with suspected or established CAD with good prognostic and therapeutic potential. With a sensitivity of 0.90 and a specificity of 0.92, CCTA revealed high diagnostic accuracy for coronary plaques compared to intravascular ultrasound (IVUS) as a reference standard, per a meta-analysis [25].

CCTA can substitute ICA in individuals with suspected acute coronary syndrome (ACS) who have a low or medium pre-test risk of CAD. When analyzing over 3000 low-risk patients with suspected ACS, four randomized controlled trials compared CCTA to the standard of therapy [26,27,28,29]. These trials confirmed what was already known about the negative predictive value of CCTA. They showed again and again that it is safe to send CCTA-negative patients home from the emergency room with a very low rate of major cardiovascular adverse events (MACE) (<1%). This reduces the time required to leave the hospital and the length of stay, saving money and allowing processes to run more smoothly. However, for patients likely to have CAD before the test, ICA should be the first imaging test because CCTA has a low negative predictive value in this group [30].

Using conventional retrospective cardiac gating approaches, the cumulative mean radiation dosage, and CCTA in adult patients varied from 6 to 20 mSv in the past (equivalent to 300–1000 chest radiographs). Incorporating prospective cardiac gating into CCTA can minimize radiation exposure by around 70% [31]. With the introduction of new generations of CT scanners, the radiation dose, contrast dose, and patient turnover time of CCTA have all lowered dramatically, while image quality has also increased. The number of layers on the multi-slice spiral CT (MSCT) scanner has been increased from 64 to 128, 256, 320, and 640. This allows for the precise measurement of the degree of coronary artery stenosis and the composition of the coronary atherosclerotic plaque. The CT coronary artery calcium score and CCTA radiation dose can now be reduced further (equal to <50 chest radiographs), and sub-millimeter accuracy can be reached with the latest 640-slice CT scanner or third-generation DSCT scanner [32]. 

Additionally, the contrast load can be decreased from an average of 80 mL to 35 mL by using these faster scanners, lowering the risk of contrast nephropathy [33,34]. Further technological advances have resulted in faster CT scanners, ranging from 640-layer dynamic-volume CT scanners to spectral CT and third-generation DSCT. The X-ray tube is the primary focus of DSCT advancement. The transition from static to rotating X-ray tubes, with improvements in its properties, such as a larger heat capacity and cooling speed, enhances the CT scanner’s efficiency and allows for a higher rack speed [35]. Using a DSCT scanner with two X-ray tubes increases the efficiency of obtaining entire data sets. Each X-ray tube must be rotated 90°, reducing the picture radiation exposure and acquisition time. The third-generation DSCT scanner can greatly boost the tube power at low potential, significantly lowering radiation exposure [35].

Accurate cross-sectional vascular information can be obtained using intravascular ultrasonography (IVUS) imaging. According to the most recent research, clinicians may accurately assess pathophysiological changes in blood vessels, illness development, and the impact of therapeutic interventions using IVUS data collected at two different times [36]. As an early indicator of arterial injury, the endothelium with osmotic dysfunction is thought to be the main factor in atherosclerosis. Tools and other methods based on magnetic resonance imaging (MRI) enable us to understand the role of endothelial permeability in cardiovascular disease and the risks in vivo [37]. The most widely used radioactive tracer in vascular research and a different marker of plaque inflammation is 18-F-fluorodeoxyglucose (18-F-FDG). Increasingly, 18-F-FDG and other PET (positron emission tomography) tracers are employed to provide imaging endpoints for cardiovascular intervention trials. Using biological processes, PET imaging can characterize the high-risk traits of susceptible atherosclerotic plaques. Inflammation, microcalcification, hypoxia, and neovascularization can all be tracked using current radioactive tracers in susceptible plaques. Developing novel PET radioactive tracers, imaging techniques, and hybrid scanners may improve the effectiveness and accuracy of characterizing high-risk plaques [38]. Plaque features are identified through a novel atherosclerosis identification approach. Plaque detection frequently uses multi-mode/hybrid imaging systems and near-infrared fluorescence imaging. In both clinical and experimental settings, Indocyanine Green (ICG) targets human plaques with endothelial anomalies and offers fresh insights into its targeting mechanism [39].

## 3. Gene and Protein Markers

### 3.1. Gene Level

**Figure 2:**
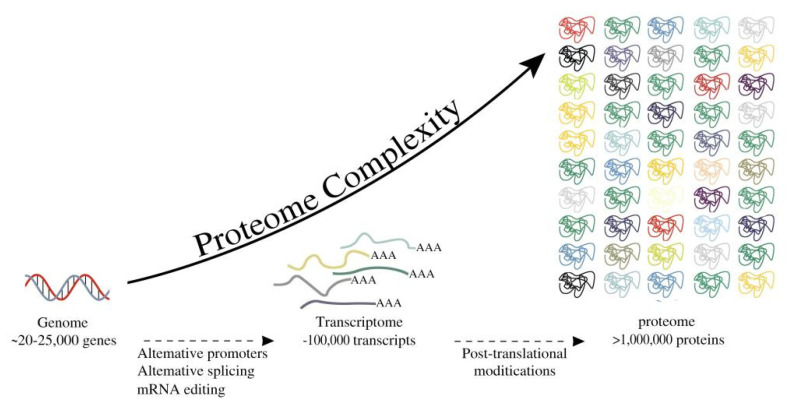
The entire process from gene to protein.

MicroRNA (miRNA), which plays an important role in regulating pathophysiological processes such as cell adhesion, proliferation, lipid uptake, efflux, and the production of inflammatory mediators, offers a new molecular understanding for investigating their effects on these pathways in coronary atherosclerosis and helps to pinpoint potential therapeutic approaches. MiRNA’s potential as a diagnostic, prognostic, or therapy response biomarker for cardiovascular disease has been particularly increased by the realization that miRNA may be detected outside of cells, even in circulating blood [40]. Figure 2 illustrates the connection between genes and proteins.

Cholesterol homeostasis is essential to the physiology of the cell. Variations in cellular or systemic cholesterol concentrations are linked to metabolic disorders. In circulation, cholesterol is transported by lipoproteins, which maintain cholesterol homeostasis by transferring (such as low-density lipoprotein (LDL)) and removing (such as high-density lipoprotein (HDL)) cholesterol from cells and tissues. High-level low-density lipoprotein cholesterol (LDLC) and/or low-level high-density lipoprotein cholesterol (HDLC) imbalances that encourage cell cholesterol buildup can induce coronary atherosclerosis. Recent discoveries of genes that regulate the abundance and function of low-density lipoprotein (LDL) and high-density lipoprotein (HDL) have significantly increased our knowledge of the regulatory circuits that regulate plasma lipoprotein levels [4,5,11].

MiRNA regulates lipoprotein metabolism and associated diseases such as metabolic syndrome, obesity, and atherosclerosis [41]. miR-33 regulates macrophage activation and mitochondrial metabolism. Furthermore, recent research has indicated that miR-33 controls vascular homeostasis and cardiac responsiveness to pressure stress. Aside from miR-33 and miR-122, single-nucleotide polymorphisms near the miRNA gene were linked to abnormal levels of human circulation lipids. Some of these miRNAs, such as miR-148a and miR-128-1, target proteins involved in cellular cholesterol metabolisms, such as the low-density lipoprotein receptor (LDLR) and the ATP binding cassette A1 (ABCA1) [42].

MiR-122 is a microRNA implicated in the metabolism of lipoproteins, and its expression is substantially enriched in the liver [43]. MiR-122 is a critical regulator of cholesterol and fatty acid production and hence a crucial regulator of lipoprotein homeostasis, as shown by tests in mice and non-human primates, where its function was inhibited [43,44]. It should be noted that miR-122 acts on specific genes in hepatocytes, rather than participating in all lipid metabolism pathways [45]. MiR-223 and miR-27b, on the other hand, as major post-transcriptional regulatory centers, regulate the gene network of cholesterol and lipoprotein metabolism [46,47]. MiR-223 suppresses the hmgs1, sc4mol, and srb1 genes involved in HDL absorption and cholesterol production, resulting in higher levels of HDL-C and total cholesterol in the liver and plasma in miR-223 mice [46].

Coronary atherosclerosis can easily occur on the artery wall due to ongoing hyperlipidemia and fluctuating shear stress. Endothelial cells experience several molecular and cellular conformational changes in response to biomechanical and biochemical stimuli, aiding coronary atherosclerosis development. For instance, leukocyte migration to the arterial wall, which may be one of the primary indicators connected to new plaques, is aided by the early elevation of the expression of adhesion molecules such as vascular adhesion molecule (VCAM)-1, intracellular adhesion molecule (ICAM)-1, and E-selectin [48]. Some miRNAs can directly target the 3′-UTR of these molecules as a result of miR-17-3p (targeting ICAM-1) and miR-31 (targeting E-selectin), which are connected to coronary atherosclerosis [49]. These molecules induce an increase in macrophages in the process of atherosclerosis [50,51,52]. It is unclear how these two miRNAs function in experimental coronary atherosclerosis. In addition to these molecules that promote adhesion, several other pro-inflammatory and pro-thrombotic factors are also activated by nuclear factor (NF)-κB signaling, which is a significant route. Two cytokine-reactive miRNAs, miR-181b and miR-146a, control NF-κ. Different components of the B signal have a protective effect on coronary atherosclerosis [53]. 

Elevated plasma levels of miR-146a-5p and miR-21-5p have been established in studies as general biomarkers of ACS circulation [54]. According to Amanpreet et al., the most prevalent miRNAs in CAD (miR-1, miR-133a, miR-208a, and miR-499) are significantly expressed in the heart and have an important role in cardiac physiology [55]. Even though studies found that numerous miRNAs are expressed in ACS, and stable CAD, miR-1, miR-133, miR-208a, and miR-499 are typically considered ACS biomarkers [41], these biomarkers, particularly miR-499, whose concentration gradient level is associated with myocardial damage, are most likely to diagnose ACS and stable CAD [55,56].

Ariana et al.’s study demonstrates that miR-132 is both required and sufficient to cause the formation of pathogenic cardiomyocytes, a hallmark of unfavorable cardiac remodeling. As a result, miR-132 can be employed as a therapeutic target for heart failure (HF). At the same time, anti-miR-132 therapy demonstrated good pharmacokinetics, safety, tolerability, a dose-dependent PK/PD relationship, and high clinical promise [39,57]. Several pathologic cellular effects and molecular signaling pathways relevant to atherosclerosis are continuously regulated and fine-tuned by miRNA [40]. The progression and balance of atherosclerotic plaques are regressed due to changes in these pathways—for instance, ventricular hypertrophy (miR-208 and miR-133), fibrosis (miR-21 and miR-29), and ventricular arrhythmias (miR-1, miR-328, and miR-133) [58]. Table 1 describes the miRNAs mentioned above.

There are potential drawbacks of using miRNA to identify atherosclerosis, including differences in the reliability of different screening methods [59]. The challenge of isolating miRNA using conventional RNA reagents necessitates the optimization of miRNA isolation from complex materials. Detection methods vary as well, with Qubit and microRNA assays offering the lowest variation (%CV 5.47, SEM ± 0.07), followed by Nano Drops (%CV 7.01, SEM ± 0.92) and the Agilent Biological Analyzer (%CV 59.21, SEM ± 1.31) [59]. The long-term clinical use of miRNAs necessitates additional work to address current methodological, technical, or analytical shortcomings. Standard operating protocols, coordinated miRNA isolation, and quantification techniques are necessary to increase repeatability among different investigations [60].

The advancement of genome-wide analysis, particularly microarray analysis, is critical in identifying clinical indicators of coronary atherosclerosis [61,62]. Whole-blood gene expression profiles can reveal illness status dynamics and suggest putative disease causes [63]. Many prevalent illnesses, such as AMI [64,65,66,67] and various forms of atherosclerosis [68], have distinct gene expression profiles. Differential gene expression in peripheral blood cells can provide more information on disease dynamics and better forecast the likelihood of cardiovascular events than currently employed approaches [63]. Changes in gene expression in peripheral blood cells have high sensitivity and specificity for diagnosing coronary heart disease (CAD) [69]. The expression level of the adior2 gene, for example, is linked to the advancement of coronary atherosclerosis [70]. Meng et al. revealed that numb ABCB1, ACSL1, ZHHC9, and other genes have important roles in the pathogenesis of atherosclerosis [71,72,73,74]. Furthermore, a study from the University of Washington discovered that the SVEP1 gene causes atherosclerosis in the absence of cholesterol [75].

### 3.2. Protein Levels

Protein is the stage following the gene level and the product of gene translation. Coronary atherosclerosis develops due to a complex combination of environmental and hereditary variables. According to recent research, smoking and stress can quickly lead to cardiovascular disease [76,77]. While genetic variables are uncontrollable, adjustments in certain environmental effects, such as lifestyle and smoking behaviors, may alleviate cardiovascular symptoms [78,79]. It is important to note that genetic factors account for 50% of the risk of atherosclerosis. As a result, early patient diagnosis using reliable genetic indicators of atherosclerosis can result in prompt and precise therapy choices. Therefore, finding new molecular markers is crucial in coronary heart disease for early detection, prompt warning, early intervention, and improved prognosis [80,81]. APOC3 and APOC4 have been confirmed to be involved in the process of atherosclerosis [82,83,84]. Meng et al. found that different proteins were present in different types of coronary atherosclerosis and that different protein markers identified different phases of atherosclerosis. Six genes (*ALB, SHBG, APOC, APOC3, APOC4*, and *SAA4*) were found to be responsible for its regulation [68].

## 4. Trace Elements

### 4.1. Zinc Ion

The rise in patients with coronary heart disease in the United States, Europe, and China is related to diet-associated raised blood cholesterol and blood glucose levels, as well as poor lifestyle habits such as smoking and genetic factors [85]. Smoking, blood sugar, lipids, and hypertension are the four main risk factors for coronary heart disease. These four independent risk variables were found to be primary predictors of coronary atherosclerosis [86,87,88]. Simultaneously, an intriguing relationship has surfaced. The prevalence of coronary heart disease in underdeveloped nations is positively connected with the human development index.

In contrast, it is inversely correlated with the human development index in developed countries (ρ = 0.47 and 0.34, accordingly). Furthermore, the incidence of coronary heart disease has increased in emerging nations over the last few decades, while it has decreased (*p* = 0.021 and 0.002) in developed countries [89,90,91,92]. This is due to dietary imbalances and differences in the serum concentrations of several trace elements [93]. 

As a result, it is worthwhile to investigate the differences in trace element concentrations in the human body and their associations with coronary heart disease. An analysis reveals that coronary heart disease and other diseases are associated with trace elements in the body [94,95,96]. Zinc ion helps to control many cellular metabolic processes, such as how proteins, lipids, and carbohydrates are broken down and used by the body [97,98]. Zinc is a crucial component of over seventy enzymes, including superoxide dismutase and glutathione peroxidase. As a cofactor of copper-zinc superoxide dismutase (Cu, Zn SOD), zinc can influence CD. Research has demonstrated that zinc supplementation can lower the activity of copper-zinc superoxide dismutase due to an antagonistic relationship between excessive zinc consumption and copper absorption [99]. Zinc also has anti-inflammatory and antioxidant effects [94]. An increased zinc concentration enhances cell antioxidant capability and ensures the maintenance of appropriate endothelium function. Due to zinc’s involvement in enzymes, humoral mediators, and mitosis, the immune system relies on zinc to function. Zinc deficiency is associated with sensitivity to oxidative stress, IL-1 and tumor necrosis factor expression, and endothelial cell death [94]. These factors are all involved in atherosclerosis progression. A decline in zinc ion concentration is associated with coronary heart disease in non-smoking older patients and women, particularly postmenopausal women. Patients with coronary artery disease benefit from taking zinc ions in the appropriate amounts [100].

### 4.2. Iron Ion

Iron is required for numerous physiological activities. Iron-containing proteins and enzymes serve as an essential part of cellular metabolism. These enzymes and proteins are essential for cell proliferation, cell death, DNA synthesis, DNA repair, and mitochondrial function [101,102,103]. Iron is the principal component of hemoglobin, which is needed to produce red blood cells and transfer oxygen. Iron is also potentially harmful in high concentrations due to its tendency to produce reactive oxygen species (ROS) and damage biomolecules via Fenton reaction-generated hydroxyl radicals [104]. It is also a significant component in determining bacterial toxicity [92].

Iron consumption or outflow irregularities can result in disease. Iron was originally implicated in coronary atherosclerosis development [104,105]. Low-density lipoprotein oxidation can be accelerated by free iron [106]. LDL receptors on macrophages then absorb LDL, causing foam cells to be recruited. Foam cell infiltration and necrotic core enlargement are crucial steps in coronary atherosclerosis development [107]. In atherosclerotic plaques, many macrophage subtypes have been identified [108]. Macrophages have a significant role in the progression of coronary atherosclerosis. Lipid absorption, which can cause the production of inflammatory cytokines and the formation of foam cells, is the principal cause of M1 macrophage activation in plaques [109]. M1 macrophages are considered to induce coronary atherosclerosis by paracrine stimulating SMC migration and proliferation from the middle membrane to the intima.

Hydrolyzing collagen fibers in the fiber cap, MMP-1, MMP-3, and MMP-9 produced by M1 cells might cause plaque instability [110]. In addition, Th2 cytokines (such as IL-4, IL-10, and IL-13) activate M2 macrophages to create anti-inflammatory cytokines. The inflammatory response is assumed to be balanced by M2 macrophages, which also support tissue repair and inflammation remission. The M1/M2 model offers a condensed structure for comprehending macrophage behavior in a damaged environment. While M2 macrophages can export and metabolize iron, M1 macrophages have high ferritin content and are superior in terms of iron accumulation. Coronary atherosclerosis may result from the variation in the iron turnover rate between M1 and M2 macrophages. The relationship between the peripheral blood iron concentration and coronary atherosclerosis was validated by a cross-sectional study involving more than 4000 individuals. Loss of peripheral blood iron ions can be used as a biomarker for coronary atherosclerosis prognosis [64].

### 4.3. Other Trace Elements

Trace elements significantly influence cardiovascular disease by directly or indirectly altering the circulatory process [111,112,113]. Blood metal levels and childhood and adolescent obesity have been demonstrated to correlate positively, according to research by Fan et al. [114]. It was discovered that obesity was associated with an increase in superoxide dismutase (SOD) levels and total circulation copper concentrations. Metal ions influence the expression of leptin in adipocytes by regulating the release of free fatty acids and glucose uptake, highlighting that obesity is a significant coronary heart disease risk factor [114,115]. As per Kalita et al., variations in trace elements can improve insulin resistance in people with type 2 diabetes [116]. Numerous diabetes-related enzymes utilize magnesium and manganese as cofactors. Their insufficiency raises the risk of metabolic syndrome, impairs glucose metabolism, and may lead to atherosclerosis [116,117]. The serum selenium level was substantially linked with all-cause mortality in both men and women, particularly women with coronary heart disease, according to Li et al. [118]. Consequently, alterations in trace element concentrations in the body are regarded as the most important factor in the development of some diseases and in transitioning from health to illness.

This article has certain limitations. The study only discusses imaging, genes and proteins, and trace elements related to atherosclerosis, but other facets of the disease should also be examined. The key to the early detection of atherosclerosis is the combination of more cutting-edge diagnostic procedures and various examination techniques.

## 5. Conclusions

It is viable to assess coronary atherosclerosis risk using genes and trace elements. In patients with definite symptoms of coronary heart disease, it is reasonable to perform noninvasive investigations such as CCTA. One of the therapy methods for coronary artery disease is the detection of trace elements, which is important for prognosis.

## Figures and Tables

**Table 1:** Description of miRNAs.

Name	miR-122 miR-223 miR-27 miR-33 miR-128 miR-148a	miR-17 miR-31	miR-181 miR-146	miR-146 miR-21 miR-1 miR-133a miR-208a miR-499	miR-132	miR-1 miR-133 miR-328	miR-21 miR-29	miR-208 miR-133
Role	Lipid metabolism	Inflammatory	Proliferation and differentiation	ACS	Heart failure	Arrhythmias	Fibrosis	Ventricular hypertrophy

## Data Availability

Not applicable.

## Abbreviation

LDL	Low-density lipoprotein
ICA	Invasive coronary angiography
CCTA	Computed tomography coronary angiography
CAD	Coronary artery disease
ACS	Acute coronary syndrome
MACE	Major cardiovascular adverse events
MSCT	Multi-slice spiral CT
DSCT	Dual-source computed tomography
IVUS	Intravascular ultrasound
MRI	Magnetic resonance imaging
18-F-FDG	18 -F-fluorodeoxyglucose
PET	Positron emission tomograph
NIR	Near-infrared imaging
ICG	Indocyanine green
miRNA	MicroRNA
CV	Coefficient of variation
ABCA1	ATP binding cassette A1
HDL	High-density lipoprotein
LDLC	Low-density lipoprotein cholesterol
HDLC	High-density lipoprotein cholesterol
VCAM	Vascular adhesion molecule
ICAM	Intracellular adhesion molecule
NF	Nuclear factor
ACS	Acute coronary syndrome
HF	Heart failure
PK/PD	Pharmacokinetics/pharmacodynamics
AMI	Acute myocardial infarction
ROS	Reactive oxygen species
SOD	Superoxide dismutase

## References

[1] Ross R. (1999). Atherosclerosis—An inflammatory disease. N. Engl. J. Med..

[2] Kanter J.E., Kramer F., Barnhart S., Averill M.M., Vivekanandan-Giri A., Vickery T., Li L.O., Becker L., Yuan W., Chait A. (2012). Diabetes promotes an inflammatory macrophage phenotype and Atherosclerosis through acyl-CoA synthetase 1. Proc. Natl. Acad. Sci. USA.

[3] Li J.J., Chen J.L. (2005). Inflammation may be a bridge connecting hypertension and Atherosclerosis. Med. Hypotheses.

[4] Falk E. (2006). Pathogenesis of Atherosclerosis. J. Am. Coll. Cardiol..

[5] Miname M.H., Santos R.D. (2019). Reducing cardiovascular risk in patients with familial hypercholesterolemia: Risk prediction and lipid management. Prog. Cardiovasc. Dis..

[6] Summerhill V.I., Grechko A.V., Yet S.F., Sobenin I.A., Orekhov A.N. (2019). The atherogenic role of circulating modified lipids in Atherosclerosis. Int. J. Mol. Sci..

[7] Taleb S. (2016). Inflammation in Atherosclerosis. Arch. Cardiovasc. Dis..

[8] Cheng F., Torzewski M., Degreif A., Rossmann H., Canisius A., Lackner K.J. (2013). Impact of glutathione peroxidase-1 deficiency on macrophage foam cell formation and proliferation: Implications for atherogenesis. PLoS ONE.

[9] Falk E., Nakano M., Bentzon J.F., Finn A.V., Virmani R. (2013). Update on acute coronary syndromes: The pathologists’ view. Eur. Heart J..

[10] Rognoni A., Cavallino C., Veia A., Bacchini S., Rosso R., Facchini M., Secco G.G., Lupi A., Nardi F., Rametta F. (2015). Pathophysiology of atherosclerotic plaque development. Cardiovasc. Hematol. Agents Med. Chem..

[11] Stary H.C., Chandler A.B., Glagov S., Guyton J.R., Insull W., Rosenfeld M.E., Schaffer S.A., Schwartz C.J., Wagner W.D., Wissler R.W. (1994). A definition of Atherosclerosis’s initial, fatty streak, and intermediate lesions. A report from the Committee on Vascular Lesions of the Council on Arteriosclerosis, American Heart Association. Circulation.

[12] Virmani R., Kolodgie F.D., Burke A.P., Farb A., Schwartz S.M. (2000). Lessons from sudden coronary death: A comprehensive morphological classification scheme for atherosclerotic lesions. Arterioscler. Thromb. Vasc. Biol..

[13] Yahagi K., Kolodgie F.D., Lutter C., Mori H., Romero M.E., Finn A.V., Virmani R. (2017). Pathology of human coronary and carotid artery atherosclerosis and vascular calcification in Diabetes mellitus. Arterioscler. Thromb. Vasc. Biol..

[14] Wang X., Matsumura M., Mintz G.S., Lee T., Zhang W., Cao Y., Fujino A., Lin Y., Usui E., Kanaji Y. (2017). In vivo calcium detection by comparing optical coherence tomography, intravascular ultrasound, and angiography. JACC Cardiovasc. Imaging.

[15] Miller J.M., Rochitte C.E., Dewey M., Arbab-Zadeh A., Niinuma H., Gottlieb I., Paul N., Clouse M.E., Shapiro E.P., Hoe J. (2008). Diagnostic performance of coronary angiography by 64-row CT. N. Engl. J. Med..

[16] Assante R., Klain M., Acampa W. (2019). Use coronary artery calcium scanning as a triage for invasive coronary angiography. J. Nucl. Cardiol..

[17] Morris P.D., Gunn J.P. (2017). Computing Fractional Flow Reserve From Invasive Coronary Angiography: Getting Closer. Circ. Cardiovasc. Interv..

[18] Minhas A., Dewey M., Vavere A.L., Tanami Y., Ostovaneh M.R., Laule M., Rochitte C.E., Niinuma H., Kofoed K.F., Geleijns J. (2019). Patient Preferences for Coronary CT Angiography with Stress Perfusion, SPECT, or Invasive Coronary Angiography. Radiology.

[19] National Research Council (2006). Health Risks from Exposure to Low Levels of Ionizing Radiation: BEIR VII Phase 2.

[20] Collet J.P., Thiele H., Barbato E., Barthelemy O., Bauersachs J., Bhatt D.L., Dendale P., Dorobantu M., Edvardsen T., Folliguet T. (2020). 2020 ESC Guidelines for the management of acute coronary syndromes in patients presenting without persistent ST-segment elevation. Eur. Heart J..

[21] Collet C., Onuma Y., Andreini D., Sonck J., Pompilio G., Mushtaq S., La Meir M., Miyazaki Y., de Mey J., Gaemperli O. (2018). Coronary computed tomography angiography for heart team decision-making in multivessel coronary artery disease. Eur. Heart J..

[22] Mushtaq S., Andreini D., Pontone G., Bertella E., Bartorelli A.L., Conte E., Baggiano A., Annoni A., Formenti A., Trabattoni D. (2014). Prognostic value of coronary CTA in coronary bypass patients: A long-term follow-up study. JACC Cardiovasc. Imaging.

[23] National Institute for Health and Clinical Excellence (2010). Chest Pain of Recent Onset: Assessment and Diagnosis of Recent Onset Chest Pain or Discomfort of Suspected Cardiac Origin.

[24] Taylor A.J., Cerqueira M., Hodgson J.M., Mark D., Min J., O’Gara P., Rubin G.D., American College of Cardiology Foundation Appropriate Use Criteria Task Force, Society of Cardiovascular Computed Tomography, American College of Radiology (2010). ACCF/SCCT/ACR/AHA/ASE/ASNC/NASCI/SCAI/SCMR 2010 appropriate use criteria for cardiac computed tomography. A report of the American College of Cardiology Foundation Appropriate Use Criteria Task Force, the Society of Cardiovascular Computed Tomography, the American College of Radiology, the American Heart Association, the American Society of Echocardiography, the American Society of Nuclear Cardiology, the North American Society for Cardiovascular Imaging, the Society for Cardiovascular Angiography and Interventions, and the Society for Cardiovascular Magnetic Resonance. J. Cardiovasc. Comput. Tomogr..

[25] Voros S., Rinehart S., Qian Z., Joshi P., Vazquez G., Fischer C., Belur P., Hulten E., Villines T.C. (2011). Coronary atherosclerosis imaging by coronary CT angiography: Current status, correlation with intravascular interrogation and meta-analysis. JACC Cardiovasc. Imaging.

[26] Goldstein J.A., Chinnaiyan K.M., Abidov A. (2011). CT-STAT Investigators The CT-STAT (Coronary Computed Tomographic Angiography for Systematic Triage of Acute Chest Pain Patients to Treatment) trial. J. Am. Coll. Cardiol..

[27] Litt H.I., Gatsonis C., Snyder B., Singh H., Miller C.D., Entrikin D.W., Leaming J.M., Gavin L.J., Pacella C.B., Hollander J.E. (2012). CT angiography for safe discharge of patients with possible acute coronary syndromes. N. Engl. J. Med..

[28] Hoffmann U., Truong Q.A., Schoenfeld D.A. (2012). ROM ICAT-II Investigators Coronary CT angiography versus standard evaluation in acute chest pain. N. Engl. J. Med..

[29] Hamilton-Craig C., Fifoot A., Hansen M., Pincus M., Chan J., Walters D.L., Branch K.R. (2014). Diagnostic performance and cost of CT angiography versus stress ECG—A randomized prospective study of suspected acute coronary syndrome chest pain in the emergency department (CT-COMPARE). Int. J. Cardiol..

[30] Arbab-Zadeh A., Miller J.M., Rochitte C.E., Dewey M., Niinuma H., Gottlieb I., Paul N., Clouse M.E., Shapiro E.P., Hoe J. (2012). Diagnostic accuracy of computed tomography coronary angiography according to pre-test probability of coronary artery disease and severity of coronary arterial calcification. The CORE-64 (coronary artery evaluation using 64-row multidetector computed tomography angiography) International Multicenter Study. J. Am. Coll. Cardiol..

[31] Kuchynka P., Lambert L., Černý V., Marek J., Ambrož D., Danek B.A., Linhart A. (2015). Coronary CT angiography. Cor Vasa.

[32] Halliburton S.S., Abbara S., Chen M.Y., Gentry R., Mahesh M., Raff G.L., Shaw L.J., Hausleiter J., Society of Cardiovascular Computed Tomography (2011). SCCT guidelines on radiation dose and dose-optimization strategies in cardiovascular CT. J. Cardiovasc. Comput. Tomogr..

[33] Zhang F., Yang L., Song X., Li Y.N., Jiang Y., Zhang X.H., Ju H.Y., Wu J., Chang R.P. (2016). Feasibility study of low tube voltage (80 kVp) coronary CT angiography combined with contrast medium reduction using iterative model reconstruction (IMR) on standard BMI patients. Br. J. Radiol..

[34] Raju R., Thompson A.G., Lee K., Precious B., Yang T.H., Berger A., Taylor C., Heilbron B., Nguyen G., Earls J. (2014). Reduced iodine load with CT coronary angiography using dual-energy imaging: A prospective randomized trial compared with standard coronary CT angiography. J. Cardiovasc. Comput. Tomogr..

[35] Ulzheimer S., Flohr T. (2009). Multislice CT: Current Technology and Future Developments. Medical Radiology.

[36] Tsiknakis N., Spanakis C., Tsompou P., Karanasiou G., Karanasiou G., Sakellarios A., Rigas G., Kyriakidis S., Papafaklis M., Nikopoulos S. (2021). IVUS Longitudinal and Axial Registration for Atherosclerosis Progression Evaluation. Diagnostics.

[37] Lavin B., Andia M.E., Saha P., Botnar R.M., Phinikaridou A. (2021). Quantitative MRI of Endothelial Permeability and (Dys)function in Atherosclerosis. J. Vis. Exp..

[38] Sriranjan R.S., Tarkin J.M., Evans N.R., Le E.P.V., Chowdhury M.M., Rudd J.H.F. (2021). Atherosclerosis imaging using PET: Insights and applications. Br. J. Pharmacol..

[39] Verjans J.W., Osborn E.A., Ughi G.J., Calfon Press M.A., Hamidi E., Antoniadis A.P., Papafaklis M.I., Conrad M.F., Libby P., Stone P.H. (2016). Targeted Near-Infrared Fluorescence Imaging of Atherosclerosis: Clinical and Intracoronary Evaluation of Indocyanine Green. JACC Cardiovasc. Imaging.

[40] Feinberg M.W., Moore K.J. (2016). MicroRNA Regulation of Atherosclerosis. Circ. Res..

[41] Seronde M.F., Vausort M., Gayat E., Goretti E., Ng L.L., Squire I.B., Vodovar N., Sadoune M., Samuel J.-L., Thum T. (2015). Circulating microRNAs and Outcome in Patients with Acute Heart Failure. PLoS ONE.

[42] Aryal B., Singh A.K., Rotllan N., Price N., Fernández-Hernando C. (2017). MicroRNAs and lipid metabolism. Curr. Opin. Lipidol..

[43] Esau C., Davis S., Murray S.F., Yu X.X., Pandey S.K., Pear M., Watts L., Booten S.L., Graham M., McKay R. (2006). miR-122 regulation of lipid metabolism revealed by in vivo antisense targeting. Cell Metab..

[44] Elmen J., Lindow M., Schutz S., Lawrence M., Petri A., Obad S., Lindholm M., Hedtjarn M., Hansen H.F., Berger U. (2008). LNA-mediated microRNA silencing in non-human primates. Nature.

[45] Elmen J., Lindow M., Silahtaroglu A., Bak M., Christensen M., Lind-Thomsen A., Hedtjarn M., Hansen J.B., Hansen H.F., Straarup E.M. (2008). Antagonism of microRNA-122 in mice by systemically administered LNA-antimiR leads to up-regulation of a large set of predicted target mRNAs in the liver. Nucleic Acids Res..

[46] Vickers K.C., Landstreet S.R., Levin M.G., Shoucri B.M., Toth C.L., Taylor R.C., Palmisano B.T., Tabet F., Cui H.L., Rye K.A. (2014). MicroRNA-223 coordinates cholesterol homeostasis. Proc. Natl. Acad. Sci. USA.

[47] Vickers K.C., Shoucri B.M., Levin M.G., Wu H., Pearson D.S., Osei-Hwedieh D., Collins F.S., Remaley A.T., Sethupathy P. (2013). MicroRNA-27b is a regulatory hub in lipid metabolism and is altered in dyslipidemia. Hepatology.

[48] Libby P., Ridker P.M., Hansson G.K. (2011). Progress and challenges in translating the biology of Atherosclerosis. Nature.

[49] Suarez Y., Wang C., Manes T.D., Pober J.S. (2010). Cutting edge: TNF-induced microRNAs regulate TNF-induced expression of E-selectin and intercellular adhesion molecule-1 on human endothelial cells: Feedback control of inflammation. J. Immunol..

[50] Shi Z.G., Sun Y., Wang K.S., Jia J.D., Yang J., Li Y.N. (2019). Effects of miR-26a/miR-146a/miR-31 on airway inflammation of asthma mice and asthma children. Eur. Rev. Med. Pharmacol. Sci..

[51] Zhou F., Liu P., Lv H., Gao Z., Chang W., Xu Y. (2021). miR-31 attenuates murine allergic rhinitis by suppressing interleukin-13-induced nasal epithelial inflammatory responses. Mol. Med. Rep..

[52] An J.H., Chen Z.Y., Ma Q.L., Wang H.J., Zhang J.Q., Shi F.W. (2019). LncRNA SNHG16 promoted proliferation and inflammatory response of macrophages through miR-17-5p/NF-κB signaling pathway in patients with Atherosclerosis. Eur. Rev. Med. Pharmacol. Sci..

[53] Sun X., Belkin N., Feinberg M.W. (2013). Endothelial microRNAs and Atherosclerosis. Curr. Atheroscler. Rep..

[54] Zhelankin A., Stonogina D., Vasiliev S., Babalyan K., Sharova E., Doludin Y., Shchekochikhin D., Generozov E., Akselrod A. (2021). Circulating Extracellular miRNA Analysis in Patients with Stable CAD and Acute Coronary Syndromes. Biomolecules.

[55] Kaur A., Mackin S.T., Schlosser K., Wong F.L., Elharram M., Delles C., Stewart D.J., Dayan N., Landry T., Pilote L. (2020). Systematic review of microRNA biomarkers in acute coronary syndrome and stable coronary artery disease. Cardiovasc. Res..

[56] Viereck J., Thum T. (2017). Circulating Noncoding RNAs as Biomarkers of Cardiovascular Disease and Injury. Circ. Res..

[57] Foinquinos A., Batkai S., Genschel C., Viereck J., Rump S., Gyöngyösi M., Traxler D., Riesenhuber M., Spannbauer A., Lukovic D. (2020). Preclinical development of a miR-132 inhibitor for heart failure treatment. Nat. Commun..

[58] Melman Y.F., Shah R., Das S. (2014). MicroRNAs in heart failure: Is the picture becoming less miRky?. Circ. Heart Fail..

[59] Wright K., de Silva K., Purdie A.C., Plain K.M. (2020). Comparison of methods for miRNA isolation and quantification from ovine plasma. Sci. Rep..

[60] de Gonzalo-Calvo D., Pérez-Boza J., Curado J., Devaux Y., EU-CardioRNA COST Action CA17129 (2022). Challenges of microRNA-based biomarkers in clinical application for cardiovascular diseases. Clin. Transl. Med..

[61] McPherson R. (2010). Chromosome 9p21 and coronary artery disease. N. Engl. J. Med..

[62] Ardissino D., Berzuini C., Merlini P.A., Mannucci P.M., Surti A., Burtt N., Voight B., Tubaro M., Peyvandi F., Spreafico M. (2011). Influence of 9p21.3 genetic variants on clinical and angiographic outcomes in early-onset myocardial infarction. J. Am. Coll. Cardiol..

[63] Aziz H., Zaas A., Ginsburg G.S. (2007). Peripheral blood gene expression profiling for cardiovascular disease assessment. Genom. Med..

[64] Meng H., Wang Y., Ruan J., Chen Y., Wang X., Zhou F., Meng F. (2022). Decreased iron ion concentrations in the peripheral blood correlate with coronary Atherosclerosis. Nutrients.

[65] Meng H., Wang X., Ruan J., Chen W., Meng F., Yang P. (2020). High expression levels of the SOCS3 gene are associated with acute myocardial infarction. Genet. Test. Mol. Biomark..

[66] Ruan J., Meng H., Wang X., Chen W., Tian X., Meng F. (2020). Low expression of FFAR2 in peripheral white blood cells may Be a genetic marker for early diagnosis of acute myocardial infarction. Cardiol. Res. Pract..

[67] Tan B., Liu L., Yang Y., Liu Q., Yang L., Meng F. (2019). Low CPNE3 expression is associated with risk of acute myocardial infarction: A feasible genetic marker of acute myocardial infarction in patients with stable coronary artery disease. Cardiol. J..

[68] Meng H., Ruan J., Chen Y., Yan Z., Shi K., Li X., Yang P., Meng F. (2021). Investigation of specific proteins related to different types of coronary atherosclerosis. Front. Cardiovasc. Med..

[69] Elashoff M.R., Wingrove J.A., Beineke P., Daniels S.E., Tingley W.G., Rosenberg S., Voros S., Kraus W.E., Ginsburg G.S., Schwartz R.S. (2011). Development of a blood-based gene expression algorithm for assessing obstructive coronary artery disease in non-diabetic patients. BMC Med. Genom..

[70] Ikonomidis I., Kadoglou N., Tsiotra P.C., Kollias A., Palios I., Fountoulaki K., Halvatsiotis I., Maratou E., Dimitriadis G., Kremastinos D.T. (2012). Arterial stiffness is associated with increased monocyte expression of adiponectin receptor mRNA and protein in patients with coronary artery disease. Am. J. Hypertens..

[71] Meng H., Li L., Ruan J., Chen Y., Yan Z., Liu J., Li X., Mao C., Yang P. (2022). Association of Low Expression of NUMB in Peripheral Blood with Acute Myocardial Infarction. Cardiol. Res. Pract..

[72] Ruan J., Meng H., Chen Y., Yan Z., Li X., Meng F. (2022). Expression of ATP-binding cassette subfamily B member 1 gene in peripheral blood patients with acute myocardial infarction. Bioengineered.

[73] Li T., Li X., Meng H., Chen L., Meng F. (2020). ACSL1 affects Triglyceride Levels through the PPARγ Pathway. Int. J. Med. Sci..

[74] Li L., Meng H., Wang X., Ruan J., Tian X., Meng F. (2022). Low ZCCHC9 Gene Expression in Peripheral Blood May Be an Acute Myocardial Infarction Genetic Molecular Marker in Patients with Stable Coronary Atherosclerotic Disease. Int. J. Med. Sci..

[75] Jung I.H., Elenbaas J.S., Alisio A., Santana K., Young E.P., Kang C.J., Kachroo P., Lavine K.J., Razani B., Mecham R.P. (2021). SVEP1 is a human coronary artery disease locus that promotes Atherosclerosis. Sci. Transl. Med..

[76] Wirtz P.H., von Känel R. (2017). Psychological stress, inflammation, and coronary heart disease. Curr. Cardiol. Rep..

[77] Jokinen E. (2015). Obesity and cardiovascular disease. Minerva Pediatr..

[78] Khot U.N., Khot M.B., Bajzer C.T., Sapp S.K., Ohman E.M., Brener S.J., Ellis S.G., Lincoff A.M., Topol E.J. (2003). Prevalence of conventional risk factors in patients with coronary heart disease. JAMA.

[79] Jayashree S., Arindam M., Vijay K.V. (2015). Genetic epidemiology of coronary artery disease: An Asian Indian perspective. J. Genet..

[80] Anderson N.L., Anderson N.G. (2002). The human plasma proteome: History, character, and diagnostic prospects. Mol. Cell. Proteom..

[81] Smith J.G., Gerszten R.E. (2017). Emerging affinity-based proteomic technologies for large-scale plasma profiling in cardiovascular disease. Circulation.

[82] Ronsein G.E., Vaisar T., Davidson W.S., Bornfeldt K.E., Probstfield J.L., O’Brien K.D., Zhao X.-Q., Heinecke J.W. (2021). Niacin Increases Atherogenic Proteins in High-Density Lipoprotein of Statin-Treated Subjects. Arterioscler. Thromb. Vasc. Biol..

[83] Stitziel N.O., Kanter J.E., Bornfeldt K.E. (2020). Emerging Targets for Cardiovascular Disease Prevention in Diabetes. Trends Mol. Med..

[84] Zha Y., Lu Y., Zhang T., Yan K., Zhuang W., Liang J., Cheng Y., Wang Y. (2021). CRISPR/Cas9-mediated knockout of APOC3 stabilizes plasma lipids and inhibits Atherosclerosis in rabbits. Lipids Health Dis..

[85] Bray G.A., Heisel W.E., Afshin A., Jensen M.D., Dietz W.H., Long M., Kushner R.F., Daniels S.R., Wadden T.A., Tsai A.G. (2018). The science of obesity management: An Endocrine Society scientific statement. Endocr. Rev..

[86] Goff D.C., Lloyd-Jones D.M., Bennett G., Coady S., D’Agostino R.B., Gibbons R., Greenland P., Lackland D.T., Levy D., O’Donnell C.J. (2014). 2013 ACC/AHA guideline on the assessment of cardiovascular risk: A report of the American College of Cardiology/American Heart Association task force on practice guidelines. Circulation.

[87] Stone N.J., Robinson J.G., Lichtenstein A.H., Merz C.N.B., Blum C.B., Eckel R.H., Goldberg A.C., Gordon D., Levy D., Lloyd-Jones D.M. (2014). 2013 ACC/AHA guideline on the treatment of blood cholesterol to reduce atherosclerotic cardiovascular risk in adults: A report of the American College of Cardiology/American Heart Association task force on practice guidelines. J. Am. Coll. Cardiol..

[88] Lusis A.J. (2000). Atherosclerosis. Nature.

[89] Zhu K.F., Wang Y.M., Zhu J.Z., Zhou Q.Y., Wang N.F. (2016). National prevalence of coronary heart disease and its relationship with human development index: A systematic review. Eur. J. Prev. Cardiol..

[90] Gaziano T.A., Bitton A., Anand S., Abrahams-Gessel S., Murphy A. (2010). Growing epidemic of coronary heart disease in low- and middle-income countries. Curr. Probl. Cardiol..

[91] Xu Z., Yu D., Yin X., Zheng F., Li H. (2017). Socioeconomic status is associated with global diabetes prevalence. Oncotarget.

[92] Argent A.C., Balachandran R., Vaidyanathan B., Khan A., Kumar R.K. (2017). Management of undernutrition and failure to thrive in children with congenital heart disease in low- and middle-income countries. Cardiol. Young.

[93] Mardones-Santander F., Rosso P., Stekel A., Ahumada E., Llaguno S., Pizarro F., Salinas J., Vial I., Walter T. (1988). Effect of a milk-based food supplement on maternal nutritional status and fetal growth in underweight Chilean women. Am. J. Clin. Nutr..

[94] Krachler M., Lindschinger M., Eber B., Watzinger N., Wallner S. (1997). Trace elements in coronary heart disease: Impact of intensi-fied lifestyle modification. Biol. Trace Elem. Res..

[95] Rovira J., Hernández-Aguilera A., Luciano-Mateo F., Cabré N., Baiges-Gaya G., Nadal M., Martín-Paredero V., Camps J., Joven J., Domingo J.L. (2018). Trace elements and Paraoxonase-1 activity in lower extremity artery disease. Biol. Trace Elem. Res..

[96] Strain J.J. (1994). Putative role of dietary trace elements in coronary heart disease and cancer. Br. J. Biomed. Sci..

[97] Shokrzadeh M., Ghaemian A., Salehifar E., Aliakbari S., Saravi S.S., Ebrahimi P. (2009). Serum zinc and copper levels in ischemic cardiomyopathy. Biol. Trace Elem. Res..

[98] Ilyas A., Shah M.H. (2015). Abnormalities of selected trace elements in patients with coronary artery disease. Acta Cardiol. Sin..

[99] Hughes S., Samman S. (2006). The effect of zinc supplementation in humans on plasma lipids, antioxidant status and thrombogenesis. J. Am. Coll. Nutr..

[100] Meng H., Wang Y., Zhou F., Ruan J., Duan M., Wang X., Yu Q., Yang P., Chen W., Meng F. (2021). Reduced Serum Zinc Ion Concentration Is Associated with Coronary Heart Disease. Biol. Trace Elem. Res..

[101] Dev S., Babitt J.L. (2017). Overview of iron metabolism in health and disease. Hemodial. Int..

[102] Ye Q., Chen W., Huang H., Tang Y., Wang W., Meng F., Wang H., Zheng Y. (2020). Iron and zinc ions, potent weapons against multidrug-resistant bacteria. Appl. Microbiol. Biotechnol..

[103] Muckenthaler M.U., Rivella S., Hentze M.W., Galy B. (2017). A red carpet for iron metabolism. Cell.

[104] Cornelissen A., Guo L., Sakamoto A., Virmani R., Finn A.V. (2019). New insights into the role of iron in inflammation and Atherosclerosis. EBioMedicine.

[105] Eijkelkamp B.A., Hassan K.A., Paulsen I.T., Brown M.H. (2011). Investigation of the human pathogen Acinetobacter baumannii under iron limiting conditions. BMC Genom..

[106] Sullivan J.L. (1981). Iron and the sex difference in heart disease risk. Lancet.

[107] Balla G., Jacob H.S., Eaton J.W., Belcher J.D., Vercellotti G.M. (1991). Hemin: A possible physiological mediator of low-density lipoprotein oxidation and endothelial injury. Arterioscler. Thromb..

[108] Bouhlel M.A., Derudas B., Rigamonti E., Dièvart R., Brozek J., Haulon S., Zawadzki C., Jude B., Torpier G., Marx N. (2007). PPARgamma activation primes human monocytes into alternative M2 macrophages with anti-inflammatory properties. Cell Metab..

[109] Jelani Q.U., Harchandani B., Cable R.G., Guo Y., Zhong H., Hilbert T., Newman J.D., Katz S.D. (2018). Effects of serial phlebotomy on vascular endothelial function: Results of a prospective, double-blind, randomized study. Cardiovasc. Ther..

[110] Klingler K.R., Zech D., Wielckens K. (2000). Haemochromatosis: Automated detection of the two-point mutations in the HFE gene: Cys282Tyr and His63Asp. Clin. Chem. Lab. Med..

[111] Aalbers T.G., Houtman J.P. (1985). Relationships between trace elements and Atherosclerosis. Sci. Total Environ..

[112] Eshak E.S., Iso H., Yamagishi K., Maruyama K., Umesawa M., Tamakoshi A. (2018). Associations between copper and zinc intakes from diet and mortality from cardiovascular disease in a large population-based prospective cohort study. J. Nutr. Biochem..

[113] Kodali H.P., Pavilonis B.T., Schooling C.M. (2018). Effects of copper and zinc on ischemic heart disease and myocardial infarction: A Mendelian randomization study. Am. J. Clin. Nutr..

[114] Fan Y., Zhang C., Bu J. (2017). Relationship between selected serum metallic elements and obesity in children and adolescents in the U.S. Nutrients.

[115] Ades P.A., Savage P.D. (2017). Obesity in coronary heart disease: An unaddressed behavioural risk factor. Prev. Med..

[116] Kalita H., Hazarika A., Devi R. (2020). Withdrawal of high-carbohydrate, high-fat diet alters the status of trace elements to ameliorate metabolic syndrome in rats with type 2 diabetes mellitus. Can. J. Diabetes.

[117] Shi Y., Zou Y., Shen Z., Xiong Y., Zhang W., Liu C., Chen S. (2020). Trace elements, PPARs, and metabolic syndrome. Int. J. Mol. Sci..

[118] Li J., Lo K., Shen G., Feng Y.Q., Huang Y.Q. (2020). Gender difference in the association of serum selenium with all-cause and cardiovascular mortality. Postgrad. Med..

